# Distributional Characterization of CBC-Derived Inflammatory Indices in Hospitalized Patients with Schizophrenia

**DOI:** 10.3390/diagnostics16121905

**Published:** 2026-06-19

**Authors:** Murat Yalçın, Mehmet Cudi Tuncer

**Affiliations:** 1 Department of Psychiatry, Gazi Yasargil Training and Research Hospital, Diyarbakır 21070, Turkey; 2 Department of Anatomy, Faculty of Medicine, Dicle University, Diyarbakir 21280, Turkey; drcudi@hotmail.com

**Keywords:** schizophrenia, inflammation, neutrophil-to-lymphocyte ratio, systemic immune–inflammation index

## Abstract

**Background:** Increasing evidence suggests that schizophrenia may be associated with peripheral immune–inflammatory alterations, although the distributional characteristics and heterogeneity of routinely available complete blood count (CBC)-derived inflammatory indices in real-world psychiatric inpatient settings remain insufficiently characterized. The present study aimed to descriptively evaluate the distributional properties of CBC-derived inflammatory markers in hospitalized patients with schizophrenia using an exploratory panel-based analytical framework. **Methods:** We conducted a retrospective cross-sectional analysis using anonymized CBC laboratory panels obtained from hospitalized patients with schizophrenia at a tertiary psychiatric center. Following panel reconstruction and quality control procedures, 858 structurally valid CBC panels were included in the analyses. Primary inflammatory indices included neutrophil-to-lymphocyte ratio (NLR), monocyte-to-lymphocyte ratio (MLR), platelet-to-lymphocyte ratio (PLR), and systemic immune–inflammation index (SII). Descriptive distributional analyses, threshold-based prevalence estimation, Spearman correlation analyses, and exploratory unsupervised clustering procedures were performed to evaluate inflammatory variability and internal distributional patterns within the dataset. **Results:** Median NLR was 2.51 (IQR: 1.95–3.55), median MLR was 0.25 (IQR: 0.19–0.31), median PLR was 124.10 (IQR: 100.40–163.94), and median SII was 686.96 (IQR: 484.81–1045.85). Threshold-based analyses demonstrated substantial variability in inflammatory burden distributions, with 35.9% of panels showing NLR > 3 and 27.0% demonstrating SII > 1000. Correlation analyses revealed strong positive associations among NLR, PLR, and SII, whereas RDW-CV and MPV demonstrated weaker and more heterogeneous relationships with the principal inflammatory indices. Exploratory clustering analyses generated two distributional clusters, including a smaller cluster exhibiting relatively higher NLR, MLR, PLR, SII, WBC, and platelet values than the remaining panels. Female panels demonstrated significantly higher PLR and SII distributions following false discovery rate (FDR) correction. **Conclusions:** The present findings suggest that CBC-derived inflammatory indices demonstrate substantial distributional variability within this panel-based schizophrenia dataset. Although the exploratory design, absence of patient-level linkage, and lack of clinical confounder adjustment substantially limit biological interpretation, routinely available hematological inflammatory markers may still provide a pragmatic framework for descriptive characterization of inflammatory variability patterns in real-world psychiatric populations. Future patient-level longitudinal studies integrating clinical, pharmacological, and molecular variables will be necessary to determine the potential clinical relevance of inflammatory heterogeneity in schizophrenia.

## 1. Introduction

Increasing evidence suggests that schizophrenia is associated with alterations in peripheral immune–inflammatory pathways and broader immune system dysregulation [1,2,3]. Peripheral inflammatory activation characterized by cytokine alterations, changes in leukocyte subpopulations, and hematological inflammatory abnormalities has been increasingly investigated within the field of immunopsychiatry. However, inflammatory findings in schizophrenia have demonstrated substantial heterogeneity across studies, and the extent to which peripheral inflammatory alterations are uniformly distributed across schizophrenia populations remains uncertain.

In the assessment of peripheral inflammation, complete blood count (CBC)-derived inflammatory indices, particularly neutrophil-to-lymphocyte ratio (NLR), monocyte-to-lymphocyte ratio (MLR), and platelet-to-lymphocyte ratio (PLR), have attracted considerable attention because of their low cost, widespread availability, scalability, and applicability in retrospective real-world datasets. Meta-analyses and systematic reviews have reported that NLR is generally elevated in patients with schizophrenia compared with healthy controls, while NLR, MLR, and PLR have also been associated with inflammatory alterations across the broader non-affective psychosis spectrum [2,4,5]. Beyond conventional case–control comparisons, these indices have increasingly been investigated as accessible surrogate markers potentially reflecting systemic inflammatory activation and immune dysregulation in severe mental disorders.

Among CBC-derived inflammatory markers, the systemic immune–inflammation index (SII) has emerged as a composite inflammatory indicator integrating thrombocytic, neutrophilic, and lymphocytic components of peripheral immune activity. SII incorporates platelet count (PLT), absolute neutrophil count (NEUT#), and absolute lymphocyte count (LYM#), and is calculated as:

SII = PLT × NEUT#/LYM#

Compared with isolated inflammatory ratios, SII has been proposed to provide a broader representation of peripheral inflammatory status because it simultaneously incorporates multiple hematological components associated with inflammatory activation and immune regulation [4,6,7].

Nevertheless, inflammation in schizophrenia is unlikely to represent a uniform phenomenon affecting all patients similarly. Previous studies have suggested considerable variability in inflammatory marker distributions across schizophrenia cohorts, raising the possibility that peripheral inflammatory burden may be unevenly distributed within clinical populations [3,8,9]. However, most previous investigations primarily focused on mean differences between schizophrenia and control groups using isolated inflammatory markers, whereas the broader distributional characteristics and internal variability of routinely available CBC-derived inflammatory indices have remained comparatively underexplored, particularly in real-world inpatient settings.

In this context, exploratory analytical approaches examining inflammatory marker distributions, correlation structures, and internal variability patterns may provide a useful descriptive framework for evaluating inflammatory heterogeneity in schizophrenia datasets. However, given the substantial influence of potential confounding factors such as smoking, metabolic status, medication exposure, obesity, infection, and clinical severity, CBC-derived inflammatory indices should be interpreted cautiously and primarily within exploratory and hypothesis-generating contexts.

The present study was designed to descriptively characterize peripheral inflammatory burden using routinely available hemogram-derived inflammatory indices in hospitalized patients with schizophrenia. Specifically, the study aimed to evaluate distributional properties, threshold-based prevalence patterns, correlation structures, and exploratory clustering characteristics of NLR, MLR, PLR, and SII within a retrospective panel-based laboratory dataset. Rather than attempting to establish definitive biological subtypes or clinically validated inflammatory phenotypes, the analytical framework was intended to explore whether routinely available inflammatory indices demonstrated substantial internal variability across the analyzed cohort.

## 2. Materials and Methods

### 2.1. Study Design and Analytical Framework

This study was designed and reported as a retrospective panel-based cross-sectional observational analysis in accordance with the recommendations of the STROBE Initiative for observational research [10]. The primary objective of the study was to descriptively characterize the distributional properties and variability of CBC-derived inflammatory indices in hospitalized patients with schizophrenia using a real-world laboratory dataset.

Given the absence of patient-level longitudinal linkage and detailed clinical variables, the analytical framework was intentionally structured within an exploratory and hypothesis-generating context. Accordingly, the analyses primarily focused on descriptive evaluation of inflammatory marker distributions, prevalence estimation, and assessment of internal correlation patterns among routinely available hematological inflammatory markers. Exploratory unsupervised clustering procedures were additionally performed to assess whether inflammatory indices demonstrated internally heterogeneous distributional patterns within the analyzed dataset rather than to establish clinically validated biological subtypes or discrete inflammatory classifications.

### 2.2. Study Design and Data Structure

The dataset consisted of panel-level exports of complete blood count (CBC) results obtained from hospitalized patients diagnosed with schizophrenia. All laboratory measurements were retrospectively retrieved from the institutional electronic laboratory database and analyzed in anonymized form. The dataset was structured at the panel level rather than the patient level, meaning that each observation represented a CBC panel rather than a uniquely identifiable individual.

A major methodological limitation of the dataset was the absence of patient-level identifiers and temporal information, including admission dates, discharge dates, sampling times, and longitudinal follow-up records. Consequently, repeated measurements originating from the same individual could not be distinguished, and longitudinal, paired, or repeated-measures analyses could not be performed. Therefore, the present study should be interpreted strictly within an exploratory panel-based descriptive framework rather than as a patient-level longitudinal investigation.

Because patient-level linkage was unavailable, the assumption of complete statistical independence between observations may have been partially weakened, introducing a potential risk of pseudoreplication. In addition, the absence of clinical and pharmacological variables precluded adjustment for several potentially important confounding factors, including smoking status, obesity, metabolic comorbidities, medication exposure, and concurrent inflammatory or infectious conditions. Accordingly, the analytical strategy was intentionally restricted to descriptive distributional characterization, prevalence estimation, correlation structure assessment, and exploratory evaluation of internal variability patterns rather than causal inference, predictive modeling, or definitive biological subtype identification.

Despite these limitations, large-scale real-world laboratory panel datasets may still provide useful descriptive insight into the distributional characteristics and variability of routinely available hematological inflammatory markers in psychiatric inpatient populations. Accordingly, the present analyses were intended primarily as exploratory and hypothesis-generating evaluations designed to support future patient-level and clinically integrated investigations.

### 2.3. Sample Reconstruction and Inclusion Criteria

The raw dataset was originally structured in a row-based long format in which individual CBC parameters were sequentially listed rather than organized as complete panel-level observations. Therefore, a preprocessing and panel reconstruction procedure was performed prior to statistical analysis. CBC panel boundaries were identified according to the recurring and standardized order of laboratory parameters within the exported dataset, with white blood cell count (WBC) serving as the initiating variable for sequential panel segmentation. This reconstruction procedure enabled transformation of the raw laboratory export into analyzable panel-level observations containing complete hematological parameter sets.

**Figure 1:**
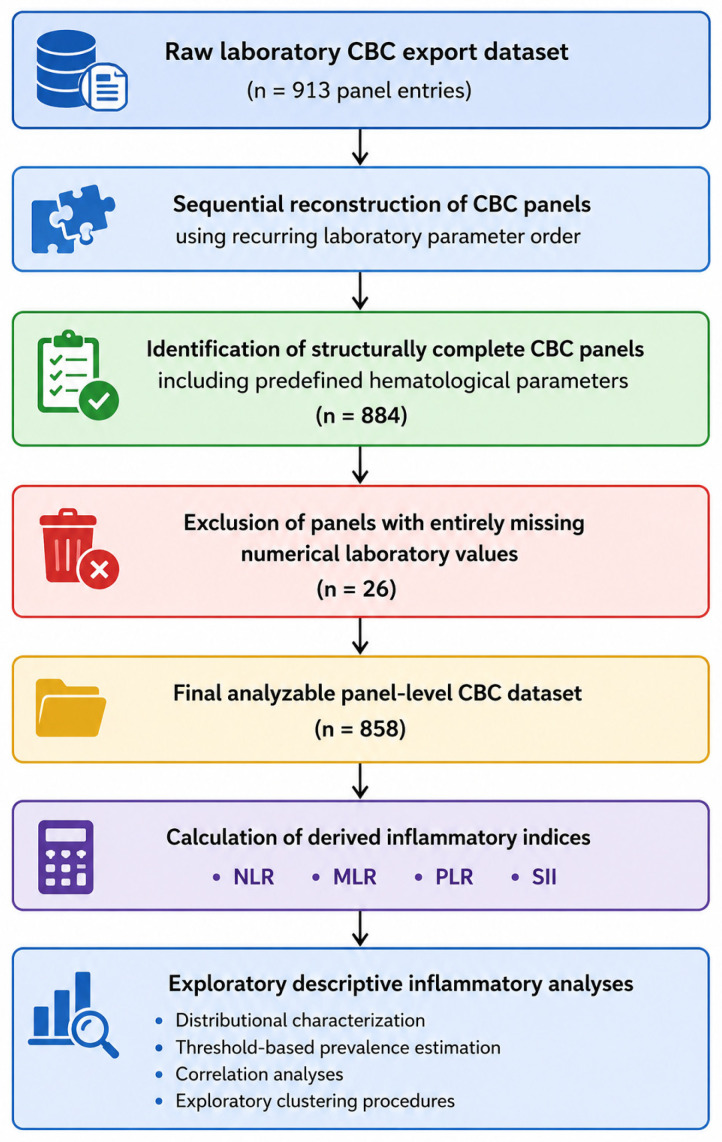
Flow diagram illustrating preprocessing, reconstruction, exclusion procedures, and final analytical selection of CBC panel data used for exploratory descriptive analyses of CBC-derived inflammatory indices in hospitalized patients with schizophrenia.

The preprocessing workflow and analytical sample selection procedures are summarized in Figure 1.

The initial dataset contained 913 CBC panel entries. Among these, 884 panels were structurally complete and included the predefined hematological parameters required for derivation of inflammatory indices and secondary hematological variables. Structural completeness was defined as the presence of the full CBC parameter sequence necessary for downstream analyses. However, 26 structurally complete panels contained entirely missing numerical laboratory values and were therefore excluded because inflammatory indices could not be reliably calculated from these observations. Consequently, the final analytical sample consisted of 858 analyzable CBC panels included in the statistical analyses.

Because the dataset was analyzed at the panel level rather than the patient level, each CBC panel was treated as an independent analytical observation within the exploratory descriptive framework. Accordingly, demographic variables including age and sex were reported descriptively at the panel level only, and no patient-level demographic inference or longitudinal interpretation was performed.

Importantly, no eligibility restrictions based on symptom severity, treatment status, hospitalization duration, medication exposure, smoking status, metabolic comorbidities, or concurrent inflammatory conditions could be applied because these variables were unavailable within the exported dataset structure. Therefore, the findings should be interpreted cautiously within a descriptive and hypothesis-generating context, particularly given the possibility that repeated measurements from the same individual may have contributed to the analyzed panel distribution.

### 2.4. Variables

#### 2.4.1. Primary Derived Inflammatory Indices

The primary analytical variables of the study consisted of CBC-derived inflammatory indices reflecting peripheral immune–inflammatory activity and hematological immune balance. These indices were calculated using routinely available absolute blood cell counts obtained from the hemogram panels.

The neutrophil-to-lymphocyte ratio (NLR) was calculated as:

NLR = NEUT#/LYM#

where NEUT# represents the absolute neutrophil count and LYM# represents the absolute lymphocyte count. NLR has been widely investigated as an accessible peripheral inflammatory marker reflecting the balance between innate inflammatory activation and adaptive immune regulation.

The monocyte-to-lymphocyte ratio (MLR) was calculated as:

MLR = MONO#/LYM#

where MONO# represents the absolute monocyte count. MLR has been proposed as a hematological marker associated with chronic inflammatory activation and monocyte-mediated immune processes.

The platelet-to-lymphocyte ratio (PLR) was calculated as:

PLR = PLT/LYM#

where PLT represents platelet count. PLR has been investigated as a peripheral inflammatory marker integrating thrombocytic activity and lymphocyte-related immune regulation.

The systemic immune–inflammation index (SII) was calculated as:

SII = (PLT × NEUT#) / LYM#

SII is a composite inflammatory marker integrating platelet count, absolute neutrophil count, and absolute lymphocyte count within a single index. Compared with isolated inflammatory ratios, SII has been proposed to provide a broader representation of peripheral inflammatory status because it simultaneously incorporates thrombocytic activity, neutrophil-associated inflammatory activation, and lymphocyte-related immune regulation [4,6,7].

These indices were selected because of their widespread availability in routine clinical practice, low computational complexity, and increasing use in exploratory immunopsychiatry research investigating peripheral inflammatory alterations in schizophrenia and related psychotic disorders. In the present study, NLR, MLR, PLR, and SII constituted the principal variables used for descriptive evaluation of inflammatory marker distributions, correlation analyses, threshold-based prevalence estimation, and exploratory clustering procedures within the panel-based analytical framework.

#### 2.4.2. Secondary Hemogram Parameters

Secondary hematological variables included white blood cell count (WBC), differential leukocyte absolute counts and percentages including neutrophils (NEUT#, NEUT%), lymphocytes (LYM#, LYM%), monocytes (MONO#, MONO%), eosinophils (EOS#, EOS%), and basophils (BASO#, BASO%). Additional platelet-related variables included platelet count (PLT), mean platelet volume (MPV), platelet distribution width (PDW), plateletcrit (PCT), and platelet-large cell ratio (P-LCR). Erythrocyte-related parameters included red blood cell count (RBC), hemoglobin (Hb), hematocrit (Hct), mean corpuscular volume (MCV), red cell distribution width coefficient of variation (RDW-CV), and red cell distribution width standard deviation (RDW-SD).

These secondary parameters were included to provide a broader descriptive characterization of hematological variation within the dataset and to support exploratory correlation analyses with the primary inflammatory indices. In particular, RDW has increasingly attracted attention as a potential hematological parameter associated with chronic inflammation, oxidative stress, altered erythropoiesis, and broader systemic physiological dysregulation. Previous studies have also reported elevated RDW levels in schizophrenia populations, suggesting that erythrocyte distribution abnormalities may accompany inflammatory or metabolic alterations within severe mental disorders [11].

Similarly, platelet-related parameters such as MPV and PDW have been investigated in psychiatric disorders as indirect indicators potentially associated with platelet activation and inflammatory activity. However, findings regarding these markers remain heterogeneous across studies and may additionally be influenced by pre-analytical variability, metabolic status, smoking, medication exposure, and other clinical confounding factors. Accordingly, MPV, PDW, and RDW-related variables were interpreted as secondary exploratory hematological parameters rather than definitive inflammatory biomarkers within the present analytical framework.

#### 2.4.3. Threshold Based Prevalence Definitions

In the present study, threshold values were used descriptively to characterize the distribution and relative magnitude of inflammatory burden within the analyzed dataset rather than to establish diagnostic or clinically validated cut-off values. Because CBC-derived inflammatory indices may be substantially influenced by demographic characteristics, smoking status, obesity, metabolic conditions, medication exposure, acute stress responses, infectious processes, and broader clinical context, universally accepted threshold values have not been established for schizophrenia populations. In particular, previous literature has emphasized that no single universal threshold exists for NLR and that reported cut-off values vary considerably across different clinical conditions and study populations [6,12].

Accordingly, prevalence analyses in the present study were performed using predefined threshold categories derived from previously reported ranges in the general inflammatory biomarker literature. Most of these threshold values were originally reported in non-psychiatric populations, particularly oncology and systemic inflammatory disease cohorts, and therefore should not be interpreted as schizophrenia-specific reference standards. These thresholds were not interpreted as disease-specific diagnostic classifiers or clinically validated inflammatory cut-offs for schizophrenia, but rather as exploratory indicators intended to illustrate relative distributional enrichment of inflammatory indices within the analyzed panel-level dataset.

The following threshold categories were used:

• NLR > 3 and >5;
• PLR > 150 and >300 [7,13];
• SII > 500 and >1000;
• RDW-CV > 14.5.

Lower threshold categories were descriptively interpreted as reflecting relatively moderate inflammatory enrichment, whereas higher thresholds represented comparatively elevated inflammatory marker distributions. Because no healthy control group or clinically validated schizophrenia-specific inflammatory reference framework was available within the present study design, prevalence estimates should be interpreted cautiously and exclusively within a descriptive exploratory context.

Reporting prevalence across multiple threshold levels was intended to facilitate evaluation of inflammatory variability and distributional heterogeneity within the dataset rather than binary classification of inflammatory status or identification of definitive inflammatory subgroups.

### 2.5. Data Processing and Quality Control

#### 2.5.1. Data Cleaning and Quality Control

Because CBC-derived inflammatory indices may be influenced by both biological variability and pre-analytical laboratory conditions, a structured data cleaning and quality control procedure was applied prior to statistical analyses. CBC measurements may be affected by factors such as sample storage duration, transportation conditions, processing delays, anticoagulant-related changes, and temperature variability. In particular, MPV and certain erythrocyte-related indices have been reported to demonstrate sensitivity to temporal and temperature-related pre-analytical variation [10,14]. Therefore, although laboratory analyses were assumed to have been performed under standardized institutional conditions, the potential influence of pre-analytical variability on selected hematological parameters was acknowledged during interpretation of the findings.

Initial preprocessing procedures included reconstruction of panel boundaries, verification of structural completeness, removal of entirely non-numerical panels, and inspection of biologically implausible values. Because CBC-derived inflammatory indices frequently demonstrate skewed distributions and occasional extreme values that may reflect either biological variability or technical variability, outlier management was performed cautiously in order to avoid artificial distortion of the overall distributional structure of the dataset.

An exploratory outlier handling strategy was implemented in two analytical stages. In the primary analyses, all observations were retained in order to preserve the original distributional characteristics of the panel-level dataset and to minimize arbitrary exclusion of potentially biologically plausible values. In secondary sensitivity analyses, observations exceeding Tukey interquartile range (IQR) criteria, defined as 1.5 × IQR beyond the lower or upper quartiles, were evaluated separately to assess robustness of descriptive findings. Where appropriate, exploratory winsorization procedures were additionally considered to reduce the disproportionate influence of extreme observations without fully excluding potentially valid laboratory measurements.

For missing data management, complete-case analysis using listwise deletion was applied because panels containing entirely missing numerical values could not support reliable calculation of derived inflammatory indices. Partial missingness within retained panels was limited and was therefore not expected to substantially influence the descriptive analytical framework of the study. However, if future patient-level datasets include larger proportions of partially missing observations and clinically integrated variables, multiple imputation by chained equations (MICE) may represent an appropriate strategy for sensitivity analyses [15,16]. In such settings, imputation of raw hematological cell counts prior to recalculation of derived inflammatory indices would be methodologically preferable in order to preserve the computational consistency of secondary variables [11].

#### 2.5.2. Potential Confounders

Several clinically relevant variables that may substantially influence peripheral inflammatory indices were unavailable within the exported laboratory dataset and therefore could not be incorporated into adjusted statistical models. The principal unavailable confounding factors included smoking status, body mass index (BMI), antipsychotic type and dosage, duration of illness, treatment resistance status, metabolic comorbidities, active infectious conditions, and additional inflammatory biomarkers such as C-reactive protein (CRP), erythrocyte sedimentation rate, cytokine profiles, or other systemic inflammatory markers.

These variables are particularly important because previous studies have demonstrated that CBC-derived inflammatory indices including NLR, PLR, MPV, and SII may be significantly affected by metabolic dysregulation, obesity-related inflammation, cigarette smoking, psychotropic medication exposure, physiological stress responses, and concurrent inflammatory or infectious processes. In schizophrenia populations specifically, antipsychotic-related metabolic alterations and chronic low-grade inflammatory activation may interact in complex ways that complicate interpretation of hematological inflammatory markers.

Because these confounding variables were unavailable, multivariable adjusted analyses controlling for potential biological and clinical confounders could not be performed. In addition, the absence of infection-related screening variables limited the ability to distinguish schizophrenia-associated inflammatory variability from non-specific inflammatory activation potentially related to concurrent medical or physiological conditions. Furthermore, the absence of a healthy control group precluded direct comparison with reference inflammatory marker distributions, limiting the ability to determine whether observed threshold enrichments reflected schizophrenia-related phenomena or broader population-level variation. Accordingly, the findings of the present study should be interpreted strictly within an exploratory descriptive framework rather than as evidence of independent or causal associations between inflammatory indices and schizophrenia-related pathophysiology.

Nevertheless, descriptive characterization of inflammatory marker distributions and internal variability patterns using routinely available hematological parameters may still provide preliminary observational insight capable of informing future patient-level, clinically integrated, and mechanistically oriented investigations.

### 2.6. Ethics, Confidentiality, and Data Security

The study was conducted using anonymized laboratory data retrospectively obtained from the institutional electronic database. Ethical approval was granted by the Clinical Research Ethics Committee of Gazi Yaşargil Training and Research Hospital, Diyarbakır, Türkiye (Approval No: 181; Date: 24 April 2026). Institutional authorization for data use was obtained in accordance with the retrospective design of the study, the absence of directly identifiable personal information, and the exclusive scientific purpose of the analyses.

Because the exported dataset did not contain patient identifiers, admission dates, national identification numbers, or other personally identifiable clinical information, individual patient confidentiality was preserved throughout all stages of data handling and statistical analysis. All analyses were performed on anonymized panel-level laboratory records under institutional data security standards and in accordance with the ethical principles of the Declaration of Helsinki.

### 2.7. Statistical Analysis Plan

Because no patient-level clinical outcome variables such as length of hospitalization, remission status, symptom severity scores, relapse history, or treatment response measures were available within the exported dataset, the statistical framework of the present study primarily focused on descriptive characterization of CBC-derived inflammatory indices within an exploratory analytical context. Accordingly, the analytical strategy was designed to evaluate the distributional properties, prevalence patterns, interrelationships, and internal variability of inflammatory markers rather than predictive clinical modeling, diagnostic classification, or causal inference analyses.

The statistical plan consisted of four principal analytical components:

1. descriptive distributional analyses of inflammatory indices and hematological variables,
2. threshold-based prevalence estimation of inflammatory marker distributions,
3. correlation structure assessment among inflammatory and hematological parameters, and
4. exploratory unsupervised clustering procedures evaluating internal variability patterns within the dataset.

Because inflammatory indices such as NLR, PLR, MLR, and SII frequently demonstrate non-normal and right-skewed distributions in clinical populations, non-parametric statistical approaches were preferentially applied where appropriate. Analytical decisions were additionally guided by the exploratory nature of the panel-level dataset and by the absence of patient-level longitudinal linkage, clinically validated outcome variables, and major confounder adjustment variables.

All statistical analyses were therefore performed within a descriptive and hypothesis-generating framework intended to characterize inflammatory variability at the panel level rather than to establish biologically discrete inflammatory subgroups, diagnostic thresholds, or predictive biomarkers.

#### 2.7.1. Descriptive Statistics and Percentile Analysis

Continuous variables were summarized using median and interquartile range (IQR) values because CBC-derived inflammatory indices and several hematological parameters demonstrated non-normal and right-skewed distributional characteristics. Distributional summaries were therefore preferentially based on robust non-parametric descriptive statistics rather than mean-based central tendency measures that may be disproportionately influenced by extreme observations.

In addition to median and IQR values, selected percentile estimates including the 1st percentile, 5th percentile, 95th percentile, and 99th percentile were calculated in order to provide a more comprehensive characterization of distributional variability and inflammatory marker dispersion within the dataset. These percentile-based summaries were intended to facilitate descriptive interpretation of lower and upper distributional extremes and to support exploratory evaluation of internal variability patterns across inflammatory indices.

Percentile tables were generated for the principal inflammatory indices including neutrophil-to-lymphocyte ratio (NLR), monocyte-to-lymphocyte ratio (MLR), platelet-to-lymphocyte ratio (PLR), and systemic immune–inflammation index (SII), as well as for selected secondary hematological variables including red cell distribution width coefficient of variation (RDW-CV) and mean platelet volume (MPV). Because no universally accepted schizophrenia-specific reference ranges currently exist for these inflammatory indices, percentile-based reporting was considered useful for contextualizing the observed distributional variability within the analyzed cohort. However, these percentile distributions should not be interpreted as clinically validated reference intervals or evidence of biologically discrete inflammatory subgroups.

#### 2.7.2. Prevalence Analysis

Threshold-based prevalence analyses were performed in order to descriptively evaluate the frequency distribution of relatively elevated inflammatory marker values within the analyzed schizophrenia dataset. Prevalence rates were reported as absolute counts and percentages using predefined threshold categories for NLR, PLR, SII, RDW-CV, and MPV. These thresholds were selected according to previously reported ranges and commonly referenced values within the broader inflammatory biomarker literature. Most were originally derived from non-psychiatric populations, particularly oncology and systemic inflammatory disease cohorts, and were used here solely for descriptive prevalence estimation rather than schizophrenia-specific classification.

The primary objective of the prevalence analyses was not to establish diagnostic classification performance, disease-specific screening utility, or clinically validated cut-off values for schizophrenia populations. Rather, prevalence estimates were intended to provide a descriptive overview of the relative distributional burden and variability of inflammatory indices within the analyzed panel-level dataset [12].

Because inflammatory indices derived from CBC parameters may be substantially influenced by demographic characteristics, medication exposure, smoking status, obesity, metabolic dysregulation, and concurrent inflammatory or infectious conditions, prevalence estimates should be interpreted cautiously and exclusively within an exploratory descriptive framework. In particular, the absence of a healthy control group and clinically integrated reference variables limited interpretation of threshold elevations as schizophrenia-specific inflammatory abnormalities.

Reporting prevalence across multiple threshold levels was therefore intended to facilitate descriptive evaluation of inflammatory variability and relative marker enrichment patterns within the dataset rather than definitive identification of relatively elevated inflammatory marker distributions within a subset of panels or clinically actionable inflammatory phenotypes.

#### 2.7.3. Correlation Analyses

Spearman rank correlation coefficient (rho) was used to evaluate associations among the primary inflammatory indices and between inflammatory indices and secondary hematological parameters. Because several CBC-derived variables demonstrated non-normal and right-skewed distributional characteristics, Spearman correlation analysis was preferred over Pearson correlation analysis due to its greater robustness against non-normality, heteroscedasticity, and extreme observations [13,17].

Correlation analyses were performed to investigate the internal relationship structure of inflammatory markers and to descriptively evaluate whether indices such as NLR, PLR, MLR, and SII demonstrated overlapping or partially distinct distributional patterns within the dataset. Additional exploratory correlations involving RDW-CV, MPV, platelet-related variables, and leukocyte subcomponents were also examined in order to characterize broader hematological interaction patterns associated with inflammatory marker variability.

Correlation strength was interpreted descriptively according to the magnitude and direction of rho coefficients. Particular emphasis was placed on evaluating coordinated distributional relationships among NLR, PLR, and SII because these markers incorporate partially overlapping hematological components associated with peripheral inflammatory activity. Accordingly, strong correlations among NLR, PLR, and SII were interpreted in part as a mathematical consequence of shared computational components rather than definitive evidence of a unified biological inflammatory axis. However, given the exploratory panel-level structure of the dataset and the absence of adjustment for major clinical confounders, correlation findings were interpreted cautiously and were not considered evidence of mechanistic or causal biological relationships.

Correlation heatmaps were additionally generated to facilitate visualization of multidimensional association patterns among inflammatory and hematological variables within the analyzed cohort.

#### 2.7.4. Group Comparisons

Group comparison analyses were performed to explore potential differences in inflammatory indices and hematological parameters across binary categorical variables available within the dataset, particularly sex-based comparisons at the panel level. Because many inflammatory and hematological variables demonstrated skewed distributions and non-normal distributional characteristics, non-parametric statistical methods were preferentially applied where appropriate.

For binary group comparisons involving non-normally distributed variables, the Mann–Whitney U test was used. Parametric independent-samples *t* tests were applied only in analyses where distributional assumptions were considered sufficiently satisfied following exploratory distribution assessment. Continuous variables were summarized according to their distributional characteristics, and both statistical significance and effect size estimates were reported in order to improve interpretability of observed group-level differences.

Because the dataset lacked clinically relevant adjustment variables including smoking status, metabolic comorbidities, medication exposure, hormonal status, and infection-related parameters, group comparison findings were interpreted cautiously within a descriptive exploratory framework. In particular, sex-related differences involving platelet-derived indices such as PLR and SII may partially reflect physiological variation in platelet counts rather than inflammation-specific biological differences.

Given the exploratory nature of the analyses and the panel-level structure of the dataset, group comparisons were not intended to establish causal biological distinctions or clinically validated subgroup classifications. For Mann–Whitney U analyses, effect size was calculated using:

$$r=\frac{\left|Z\right|}{\sqrt{N}}$$

where Z represents the standardized test statistic and N represents total sample size. For parametric comparisons, Cohen d values were reported as standardized effect size estimates.

Because multiple hematological variables and inflammatory indices were analyzed simultaneously, correction for multiple hypothesis testing was performed using the Benjamini–Hochberg false discovery rate (FDR) procedure in order to reduce the probability of false-positive findings associated with repeated statistical testing [14,18]. Adjusted q values were therefore considered alongside unadjusted *p* values during interpretation of statistical significance.

However, given the exploratory nature of the study, the panel-level structure of the dataset, and the absence of longitudinal clinical outcomes and major confounder adjustment variables, statistical significance was interpreted cautiously. Accordingly, group comparison analyses were considered primarily as descriptive evaluations of inflammatory variability rather than confirmatory evidence of independent or causal biological differences.

#### 2.7.5. Multiple Testing Correction

Because the analytical framework included evaluation of multiple hematological variables, inflammatory indices, correlation analyses, and exploratory group comparisons, correction for multiple statistical testing was performed in order to reduce the likelihood of false-positive findings arising from repeated hypothesis testing. The Benjamini–Hochberg false discovery rate (FDR) procedure was applied in the primary analyses to control the expected proportion of falsely rejected null hypotheses while preserving reasonable statistical sensitivity within the context of exploratory biomarker research [14,18].

Adjusted q values derived from the FDR procedure were reported alongside unadjusted *p* values where applicable. Statistical interpretation therefore considered both nominal significance and multiplicity-adjusted significance thresholds. The FDR approach was preferentially selected over more conservative family-wise error correction methods because the present study involved multiple correlated hematological variables and exploratory multidimensional analyses of CBC-derived inflammatory indices.

Nevertheless, given the exploratory descriptive design of the study, the panel-level structure of the dataset, and the absence of clinically integrated outcome variables and major confounder adjustment, multiplicity-adjusted statistical findings were interpreted cautiously and were not considered confirmatory evidence of biologically discrete inflammatory subgroups.

#### 2.7.6. Exploratory Clustering Analysis of Inflammatory Marker Distributions

Exploratory unsupervised clustering analyses were performed using the principal CBC-derived inflammatory indices including neutrophil-to-lymphocyte ratio (NLR), monocyte-to-lymphocyte ratio (MLR), platelet-to-lymphocyte ratio (PLR), and systemic immune–inflammation index (SII). Prior to clustering procedures, all variables were standardized using z-score normalization in order to minimize scale-related dominance effects and to ensure comparable contribution of each inflammatory parameter to cluster formation.

K-means clustering analysis was selected as an exploratory analytical approach because of its practical applicability in multidimensional biomarker datasets and its ability to evaluate internal distributional variability patterns among correlated inflammatory indices [19]. Importantly, the objective of clustering analyses in the present study was not diagnostic classification or identification of biologically validated inflammatory subgroups, but rather descriptive exploration of whether CBC-derived inflammatory indices demonstrated internally heterogeneous distributional patterns within the analyzed panel-level dataset.

The number of clusters was evaluated through integration of statistical and interpretative considerations. Statistical evaluation included examination of inertia reduction patterns using the elbow method and exploratory assessment of silhouette coefficients to estimate relative cluster separation and internal consistency. However, given the right-skewed distributional characteristics of several inflammatory indices and the exploratory structure of the dataset, clustering findings were interpreted cautiously and were not considered evidence of biologically discrete inflammatory subgroups or biologically discrete schizophrenia subtypes.

Cluster-specific inflammatory characteristics were summarized using median and interquartile range (IQR) values for NLR, MLR, PLR, SII, and selected secondary hematological parameters. Additional visualization procedures, including scatter plot-based cluster mapping, were performed to facilitate descriptive evaluation of multidimensional inflammatory marker distributions within the dataset.

Contemporary immunopsychiatry frameworks increasingly suggest that schizophrenia may involve substantial inflammatory variability rather than representing a biologically homogeneous disorder [8,11]. Nevertheless, because the present dataset lacked patient-level linkage, longitudinal structure, healthy control comparisons, and adjustment for major clinical confounding variables, clustering analyses should be interpreted exclusively within a descriptive and hypothesis-generating framework. Accordingly, identified cluster patterns were considered exploratory representations of internal inflammatory variability rather than clinically actionable or biologically validated inflammatory subgroup classifications.

**Figure 2:**
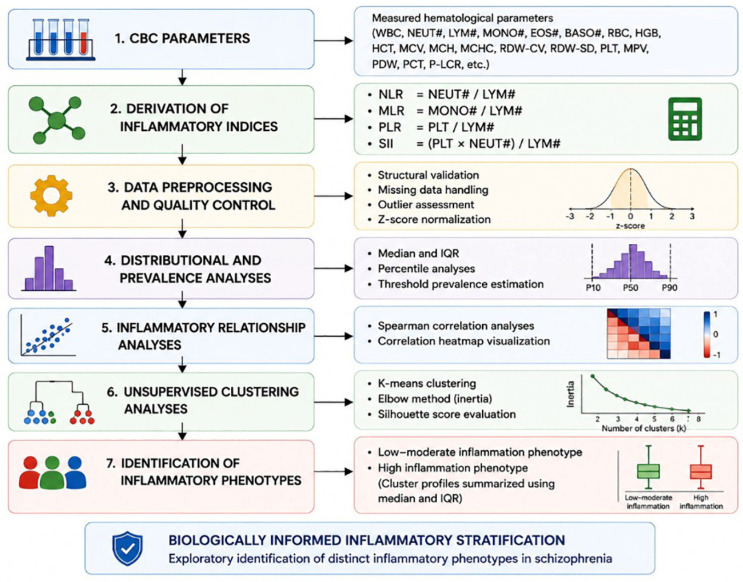
Analytical workflow illustrating derivation of CBC-based inflammatory indices, exploratory clustering procedures, and distributional characterization of inflammatory marker variability.

The overall analytical workflow used for inflammatory phenotype identification is summarized in Figure 2.

#### 2.7.7. Receiver Operating Characteristic Analysis

Receiver operating characteristic (ROC) analysis was not considered a primary analytical component of the present study because the dataset did not contain clinically defined outcome variables or validated diagnostic reference standards that could serve as ground-truth classification targets. Specifically, variables such as remission status, relapse occurrence, treatment response, symptom severity scores, prolonged hospitalization, or clinically defined inflammatory states were unavailable within the exported panel-level dataset.

Because ROC methodology is fundamentally dependent on outcome-based discrimination between predefined clinical states, the absence of patient-level outcome variables substantially limited the interpretability and clinical validity of classification performance analyses within the current study framework. Accordingly, the present analyses primarily focused on descriptive evaluation of inflammatory marker distributions and exploratory evaluation of internal variability patterns rather than predictive diagnostic modeling or clinically actionable classification approaches.

Nevertheless, CBC-derived inflammatory indices including NLR, PLR, SII, RDW-CV, and MPV have previously been evaluated using ROC-based approaches in psychiatric and inflammatory biomarker research in order to investigate potential discriminatory performance and clinically informative threshold values [11]. Therefore, if future patient-level datasets include clinically meaningful endpoints such as remission, relapse, treatment resistance, symptom severity, or hospitalization-related outcomes, ROC analyses may provide a useful framework for evaluating classification performance of inflammatory indices through calculation of area under the curve (AUC), sensitivity, specificity, and threshold estimation procedures such as the Youden index.

Within the context of the present study, ROC-related considerations were therefore discussed only as methodological perspectives relevant to future clinically integrated investigations rather than as definitive diagnostic performance analyses or evidence supporting predictive inflammatory biomarkers.

## 3. Results

### 3.1. Analytical Sample Selection and Panel Reconstruction

The initial dataset consisted of 913 CBC panels extracted from the institutional laboratory database. Following panel reconstruction procedures, 884 panels were identified as structurally complete and contained the predefined hematological parameters required for downstream analyses. Among these structurally complete panels, 26 were excluded because they lacked numerical laboratory values necessary for reliable calculation of derived inflammatory indices. Consequently, the final analytical sample consisted of 858 analyzable CBC panels included in the statistical analyses (Figure 1).

Because the dataset was structured at the panel level rather than the patient level, each analytical observation represented a CBC panel rather than a uniquely identifiable individual. Repeated measurements originating from the same patient could not be distinguished.

### 3.2. Panel-Level Demographic Characteristics

At the panel level, 525 CBC panels (61.2%) were associated with male patients, whereas 333 panels (38.8%) were associated with female patients. The median age was 32 years (IQR: 25–41 years). Because patient-level identifiers were unavailable, demographic characteristics are presented at the panel level and should not be interpreted as representing unique individual-level distributions.

### 3.3. Distributional Characteristics of Hemogram Parameters and Derived Inflammatory Indices

The distributional properties of primary hematological variables and CBC-derived inflammatory indices are summarized in Table 1. Median WBC was 8.73 (IQR: 7.22–10.58). Median absolute neutrophil count was 5.55 (IQR: 4.41–7.28), median lymphocyte count was 2.18 (IQR: 1.70–2.66), median monocyte count was 0.54 (IQR: 0.42–0.66), and median platelet count was 270 (IQR: 226.25–326.00).

Among secondary hematological parameters, median RDW-CV was 13.40 (IQR: 12.80–14.40), whereas median MPV was 10.10 (IQR: 9.40–11.00).

For the principal derived inflammatory indices, median neutrophil-to-lymphocyte ratio (NLR) was 2.51 (IQR: 1.95–3.55), median monocyte-to-lymphocyte ratio (MLR) was 0.25 (IQR: 0.19–0.31), median platelet-to-lymphocyte ratio (PLR) was 124.10 (IQR: 100.40–163.94), and median systemic immune–inflammation index (SII) was 686.96 (IQR: 484.81–1045.85) (Table 1).

Overall, inflammatory indices demonstrated substantial distributional variability across the analyzed panel-level dataset, with several variables showing broad interquartile ranges and right-skewed distributional patterns consistent with heterogeneous inflammatory marker distributions. These findings provide a descriptive overview of inflammatory marker distributions within the analyzed dataset and should not be interpreted as evidence of schizophrenia-specific inflammatory abnormalities.

Data are presented at the panel level rather than the patient level. Repeated observations originating from the same individual could not be excluded because patient-level identifiers were unavailable.

**Figure 3:**
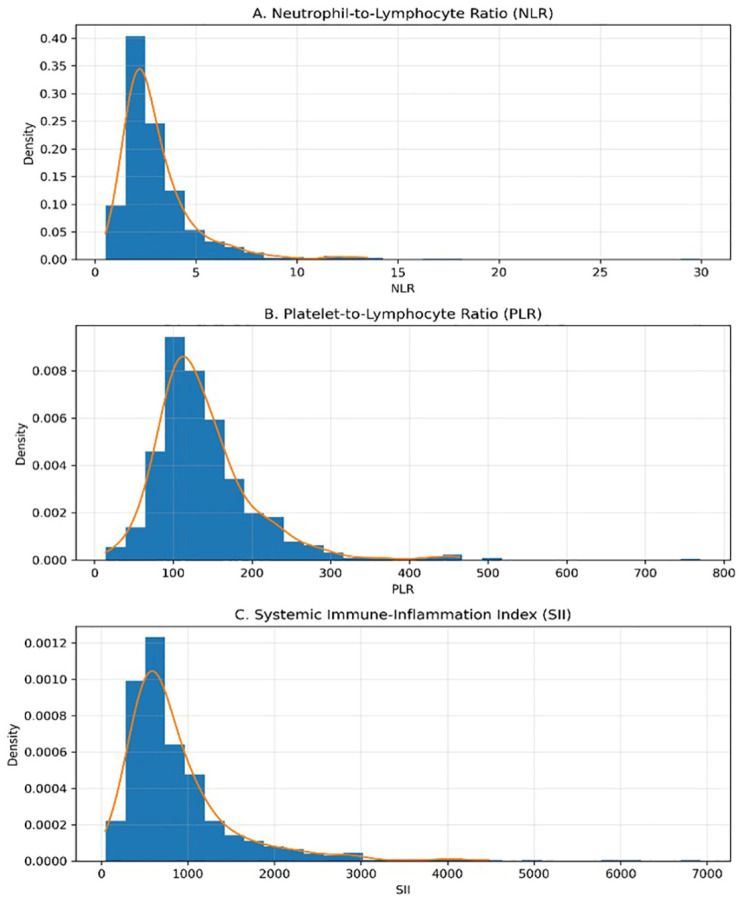
Distributional characteristics of NLR, PLR, and SII within the analyzed schizophrenia cohort. Histograms with superimposed kernel density estimation curves demonstrate non-normal and right-skewed distributions for all three inflammatory indices, particularly for PLR and SII, with extended upper-tail variability indicating substantial heterogeneity in peripheral inflammatory burden across the panel-level dataset. Panel (**A**) represents NLR distribution, Panel (**B**) represents PLR distribution, and Panel (**C**) represents SII distribution.

Visual distributional analyses demonstrated substantial right-skewed distributions and extended upper-tail variability for NLR, PLR, and SII, particularly for PLR and SII (Figure 3). These findings were consistent with considerable distributional variability of CBC-derived inflammatory indices within the analyzed panel-level dataset and further supported the use of non-parametric statistical approaches throughout the analyses.

Because inflammatory indices demonstrated broad distributional dispersion and occasional extreme values, exploratory clustering procedures were additionally considered appropriate for descriptive evaluation of internal variability patterns within the dataset. However, given the absence of patient-level linkage, healthy control comparisons, and adjustment for major clinical confounding variables, observed distributional heterogeneity should not be interpreted as definitive evidence of biologically discrete inflammatory subgroups.

### 3.4. Threshold Based Prevalence of Elevated Inflammatory Indices

Threshold-based prevalence analyses demonstrated substantial variability in the distribution of CBC-derived inflammatory indices across the analyzed panel-level schizophrenia dataset. Using predefined exploratory threshold categories, 308 panels (35.9%) demonstrated neutrophil-to-lymphocyte ratio (NLR) values greater than 3, whereas 99 panels (11.5%) exhibited NLR values greater than 5.

For platelet-to-lymphocyte ratio (PLR), values exceeding 150 were observed in 274 panels (31.9%), while values above 300 were identified in 21 panels (2.5%). Systemic immune–inflammation index (SII) values greater than 500 were present in 624 panels (72.7%), whereas 232 panels (27.0%) demonstrated SII values exceeding 1000.

Among secondary hematological parameters, RDW-CV values above 14.5 were observed in 188 panels (21.9%), and MPV values above 11 were identified in 196 panels (22.8%) (Table 2).

Overall, prevalence estimates demonstrated broad distributional variability across inflammatory marker categories. However, because threshold definitions were derived from previously reported exploratory ranges, primarily originating from non-psychiatric populations, rather than clinically validated schizophrenia-specific cut-off values, these findings should be interpreted descriptively and not as evidence of definitive inflammatory subgroup classification or clinically established inflammatory abnormality.

Collectively, these findings further supported the presence of substantial distributional variability in CBC-derived inflammatory indices within the analyzed panel-level dataset and suggested that relatively elevated inflammatory marker values were disproportionately represented within certain subsets of panels rather than being uniformly distributed across the overall dataset. However, given the exploratory panel-based design and the absence of patient-level clinical and longitudinal variables, these observations should not be interpreted as evidence of clinically validated or biologically discrete inflammatory subgroups.

### 3.5. Correlation Patterns Among Inflammatory Indices

Spearman correlation analyses demonstrated that the strongest positive associations were concentrated among neutrophil-to-lymphocyte ratio (NLR), platelet-to-lymphocyte ratio (PLR), and systemic immune–inflammation index (SII), indicating substantial overlap among the principal inflammatory indices. In particular, SII demonstrated strong positive correlations with both NLR and PLR, consistent with its composite structure integrating neutrophilic, lymphocytic, and thrombocytic hematological components. Accordingly, the strong correlations observed among NLR, PLR, and SII should be interpreted in part as a mathematical consequence of shared computational components rather than definitive evidence of a unified biological inflammatory axis.

**Figure 4:**
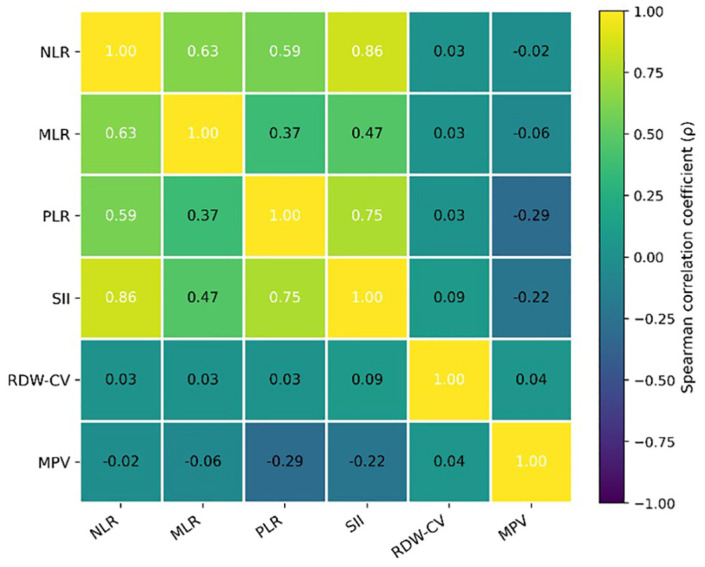
Publication-style Spearman correlation heatmap illustrating the relationships among NLR, MLR, PLR, SII, RDW-CV, and MPV. Spearman correlation coefficients (ρ) are displayed within each cell. Strong positive correlations are concentrated among NLR, PLR, and SII, supporting the presence of a shared peripheral inflammatory axis, whereas RDW-CV and MPV demonstrate comparatively weaker and more heterogeneous associations with the principal inflammatory indices.

In contrast, RDW-CV and MPV demonstrated weaker and more heterogeneous correlation patterns with the principal inflammatory indices (Figure 4). These findings suggest that NLR, PLR, and SII may reflect partially overlapping inflammatory marker distributions, whereas RDW-CV and MPV potentially represent broader and more multifactorial hematological processes influenced by additional physiological, metabolic, erythropoietic, and pre-analytical factors.

Overall, the observed correlation structure supported a descriptive distinction between inflammatory indices directly derived from leukocyte-related hematological parameters and secondary hematological variables demonstrating more heterogeneous association patterns. However, because the dataset lacked patient-level clinical variables, infection-related screening parameters, and adjustment for major confounding factors, the observed correlations should not be interpreted as evidence of mechanistic biological pathways or causal inflammatory interactions.

### 3.6. Exploratory Cluster Analysis of Inflammatory Marker Distributions

Unsupervised k-means clustering performed using z-score standardized neutrophil to lymphocyte ratio (NLR), monocyte to lymphocyte ratio (MLR), platelet to lymphocyte ratio (PLR), and systemic immune–inflammation index (SII) identified two distributional inflammatory clusters within the analyzed schizophrenia cohort.

Cluster 0, representing panels with relatively lower inflammatory marker distributions, included 715 panels (83.3%). This cluster was characterized by comparatively lower inflammatory marker distributions, with median NLR of 2.30, median MLR of 0.23, median PLR of 116.95, and median SII of 626.21.

**Figure 5:**
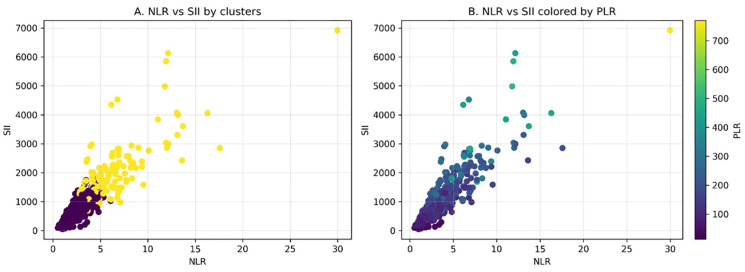
Scatter plot visualization of exploratory clustering based on CBC-derived inflammatory indices. (**A**) Distribution of NLR and SII according to k-means cluster assignment (k = 2) generated using z-score standardized NLR, MLR, PLR, and SII values. (**B**) Scatter plot of NLR versus SII with PLR represented as a continuous color gradient. The plots illustrate the distributional grouping generated by the clustering procedure, with one cluster exhibiting relatively higher NLR, PLR, and SII values than the other. These visualizations are intended to facilitate descriptive evaluation of internal variability patterns and should not be interpreted as evidence of biologically discrete inflammatory subgroups or validated inflammatory phenotypes.

In contrast, Cluster 1, representing panels with relatively elevated inflammatory marker distributions, included 143 panels (16.7%) and demonstrated substantially elevated inflammatory indices across all primary markers. Median NLR in this cluster was 5.62, median MLR was 0.40, median PLR was 222.39, and median SII was 1746.71. In addition, the higher-inflammatory cluster exhibited higher median WBC and PLT values compared with the lower-inflammatory cluster (WBC: 10.66 vs. 8.42; PLT: 310 vs. 266), further indicating that this cluster was characterized by comparatively higher inflammatory marker distributions than the remaining panels in the dataset (Table 3 and Figure 5).

The observed separation between cluster-specific inflammatory marker distributions suggested that peripheral inflammatory indices were not uniformly distributed across the analyzed panel-level dataset. In particular, a smaller subset of panels demonstrated relatively elevated values across multiple CBC-derived inflammatory indices compared with the larger cluster characterized by comparatively lower inflammatory marker distributions.

However, because clustering analyses were performed within an exploratory unsupervised framework and the dataset lacked patient-level linkage, longitudinal structure, healthy control comparisons, and adjustment for major clinical confounding variables, these findings should be interpreted cautiously. Visual inspection of the clustering structure suggested only modest separation between groups, with substantial overlap remaining across portions of the multidimensional inflammatory marker space. Accordingly, the identified clusters were considered descriptive representations of internal inflammatory variability rather than evidence of biologically discrete or clinically validated inflammatory subgroups or clinically validated schizophrenia subtypes.

### 3.7. Sex Related Differences in Inflammatory Indices

**Figure 6:**
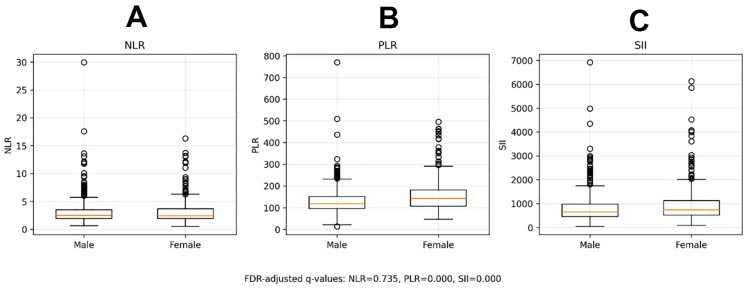
Sex-based distributions of CBC-derived inflammatory indices demonstrated using boxplot visualization. (**A**) NLR, (**B**) PLR, and (**C**) SII distributions in male and female panels. FDR-adjusted q values are displayed below the plots. Female panels demonstrated significantly higher PLR and SII distributions, whereas no statistically significant sex-related difference was observed for NLR following FDR correction.

Sex-based comparisons demonstrated no statistically significant difference in neutrophil-to-lymphocyte ratio (NLR) distributions between male and female panels following false discovery rate (FDR) correction. In contrast, platelet-to-lymphocyte ratio (PLR) and systemic immune–inflammation index (SII) demonstrated significantly higher distributions in female panels compared with male panels after multiplicity adjustment (Figure 6).

Visual inspection of boxplot distributions additionally suggested substantial intra-group variability and the presence of extreme upper-tail inflammatory values in both sexes, particularly for SII distributions.

Because PLR and SII directly incorporate platelet count within their computational structure, the observed sex-related differences may partially reflect physiological sex-related variation in platelet parameters rather than inflammation-specific biological divergence alone. Furthermore, given the absence of adjustment for smoking status, metabolic variables, medication exposure, hormonal factors, and additional clinical confounders, these findings should be interpreted cautiously within a descriptive exploratory framework rather than as definitive evidence of sex-specific inflammatory dysregulation in schizophrenia.

## 4. Discussion

Schizophrenia is increasingly conceptualized as a clinically and biologically heterogeneous disorder in which immune–inflammatory dysregulation may contribute to variability in symptom expression, disease trajectory, and broader neurobiological alterations rather than representing a uniform pathological process [20,21,22,23]. Contemporary immunopsychiatry models suggest that peripheral inflammatory alterations may interact with oxidative stress, microglial activation, blood–brain barrier dysfunction, and aberrant neurodevelopmental pathways, potentially contributing to symptom heterogeneity and treatment resistance [20,21,24]. Emerging evidence further suggests that impaired glymphatic and neuroimmune clearance mechanisms may contribute to the accumulation of inflammatory mediators, oxidative stress, and altered brain homeostasis in psychiatric disorders, providing an additional conceptual framework linking peripheral inflammatory processes with central neurobiological alterations [25]. Within this broader conceptual framework, routinely available CBC-derived inflammatory indices may provide a pragmatic and scalable approach for exploratory investigation of inflammatory variability in real-world psychiatric populations, particularly when advanced molecular or neuroimaging biomarkers are unavailable.

In the present panel-level dataset, distributions of neutrophil-to-lymphocyte ratio (NLR), platelet-to-lymphocyte ratio (PLR), and systemic immune–inflammation index (SII) demonstrated broad variability, and threshold-based prevalence analyses revealed substantial dispersion across predefined exploratory inflammatory categories (Table 1 and Table 2). These findings are generally consistent with previous meta-analyses reporting elevated NLR, MLR, and PLR values across schizophrenia and non-affective psychosis populations [2,4,5]. However, because the present study lacked healthy control comparisons, patient-level linkage, and adjustment for major clinical confounders, the observed variability should be interpreted cautiously and not as evidence of schizophrenia-specific inflammatory abnormality. Rather, the findings primarily support the descriptive observation that CBC-derived inflammatory indices are unevenly distributed across the analyzed panel-level dataset.

An important aspect of the present study is the use of a large-scale real-world laboratory dataset reflecting routine psychiatric inpatient practice rather than highly selective experimental cohorts. At the same time, the panel-based structure of the dataset imposes important methodological limitations because repeated measurements originating from the same individual could not be distinguished. Consequently, the present findings should be interpreted as exploratory descriptive observations at the panel level rather than patient-level biological inferences.

Exploratory clustering procedures additionally demonstrated that a smaller subset of panels exhibited relatively elevated NLR, MLR, PLR, and SII distributions compared with the larger cluster characterized by comparatively lower inflammatory marker levels (Table 3). Importantly, these clustering results should not be interpreted as evidence of biologically discrete or clinically validated inflammatory schizophrenia subtypes. As emphasized by previous methodological discussions, unsupervised clustering applied to right-skewed inflammatory variables may intrinsically generate relatively “higher” and “lower” distributional groupings even in the absence of true biological subgroup structure. Accordingly, the identified clusters are more appropriately interpreted as descriptive representations of internal inflammatory variability within the analyzed dataset rather than biologically discrete inflammatory subgroups.

Nevertheless, the observed clustering structure remains conceptually relevant within the broader literature suggesting that schizophrenia may involve substantial inflammatory heterogeneity [3,8,9,26,27]. Previous subgroup-oriented investigations integrating inflammatory biomarkers with neuroimaging, transcriptomic, and cognitive approaches have reported that inflammatory marker variability may be associated with broader neurobiological and clinical differences [21,27]. However, because the current dataset did not include neurocognitive variables, imaging findings, cytokine profiles, medication exposure, or longitudinal outcomes, the present analyses cannot determine whether the observed inflammatory variability corresponds to biologically meaningful or clinically actionable subgroup distinctions.

SII has increasingly been investigated as a composite marker of systemic inflammatory activity in psychiatric and non-psychiatric populations, while indices such as NLR, PLR, and SII have been associated with cognitive impairment, metabolic alterations, and broader inflammatory burden in schizophrenia-related research [28,29]. In the present study, these indices demonstrated broad distributional variability and strong internal correlations, particularly along the NLR–PLR–SII axis. However, because SII mathematically incorporates platelet count, neutrophil count, and lymphocyte count within the same formula, part of the observed intercorrelation structure is expected from the computational overlap of these variables rather than representing independent biological processes.

RDW-CV and MPV demonstrated comparatively weaker and more heterogeneous association patterns with the principal inflammatory indices. Previous studies have linked RDW to chronic inflammation, oxidative stress, impaired erythropoiesis, and cardiometabolic risk in schizophrenia populations [11]. Similarly, MPV has been investigated as an indirect marker of platelet activation and inflammatory activity, although findings remain heterogeneous across psychiatric disorders and are sensitive to methodological variation [2,30]. In addition, MPV may be substantially influenced by pre-analytical factors including sample storage duration, anticoagulant exposure, and temperature-related variability [10,14]. Accordingly, RDW-CV and MPV were interpreted cautiously as secondary exploratory hematological variables rather than definitive inflammatory biomarkers within the present analytical framework.

Sex-based analyses demonstrated significantly higher PLR and SII distributions in female panels following false discovery rate correction. However, these findings should be interpreted cautiously because both PLR and SII directly incorporate platelet count within their computational structure, and physiological sex-related differences in platelet parameters may partially contribute to the observed distributions independent of inflammation-specific mechanisms. Furthermore, important confounding variables including smoking status, obesity, metabolic syndrome, hormonal status, medication exposure, and concurrent inflammatory conditions were unavailable within the dataset.

**Figure 7:**
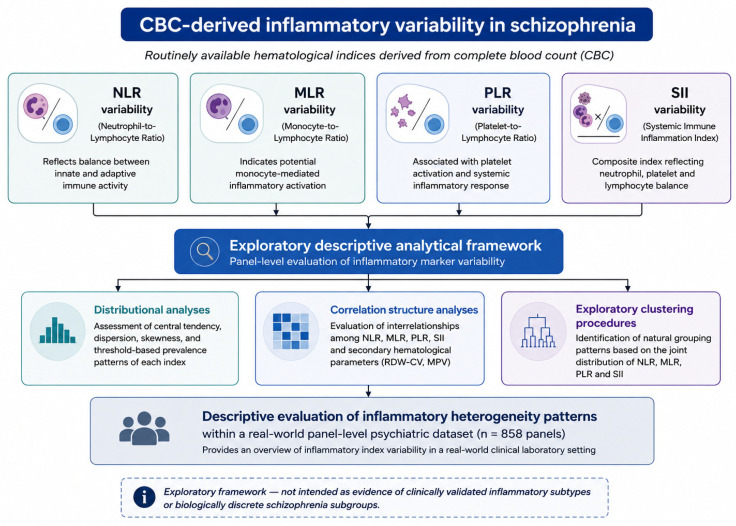
Graphical overview summarizing the distributional variability of CBC-derived inflammatory indices within the analyzed panel-level schizophrenia dataset and the exploratory analytical framework used for descriptive evaluation of inflammatory marker heterogeneity. The schematic illustrates how routinely available hematological indices may contribute to exploratory investigation of inflammatory variability patterns in real-world psychiatric populations.

The overall conceptual framework of the present study, including the distributional variability of CBC-derived inflammatory indices and the exploratory analytical framework used to evaluate inflammatory heterogeneity within the panel-level dataset, is summarized visually in Figure 7. The graphical overview illustrates how routinely available hematological inflammatory indices may contribute to descriptive investigation of inflammatory variability patterns in real-world psychiatric populations. However, the schematic representation should be interpreted within the exploratory limitations of the present study design and not as evidence of biologically discrete inflammatory subgroups or clinically validated schizophrenia subtypes.

Several additional limitations should be emphasized. The most important limitation of the present study is the absence of patient-level identifiers, which precluded determination of the number of unique individuals represented in the dataset and prevented differentiation of repeated observations originating from the same patient. Consequently, repeated measurements may have contributed to the analyzed distributions, potentially weakening the assumption of complete statistical independence and introducing pseudoreplication bias. Therefore, all prevalence estimates, correlation analyses, clustering results, and sex-based comparisons should be interpreted strictly as panel-level observations rather than patient-level findings. Second, clinically important confounding variables including smoking status, BMI, antipsychotic treatment characteristics, metabolic comorbidities, infection-related variables, and additional inflammatory biomarkers such as CRP or cytokine profiles were unavailable. Third, the absence of a healthy control group substantially limits interpretation of threshold-based inflammatory enrichment patterns. Fourth, because the dataset was cross-sectional and panel-based, longitudinal inflammatory trajectories and clinical outcome associations could not be evaluated. Finally, clustering analyses were exploratory in nature and were not externally validated using independent cohorts or multimodal biological datasets.

Despite these limitations, the present study provides a large-scale descriptive overview of CBC-derived inflammatory index distributions within a real-world psychiatric inpatient dataset. The findings support the broader concept that inflammatory marker variability in schizophrenia-related populations may be substantial and non-uniform, while also highlighting the importance of cautious interpretation when routinely available hematological biomarkers are analyzed without patient-level clinical integration. Future studies incorporating longitudinal patient-level designs, healthy control comparisons, clinically integrated outcome measures, and multimodal inflammatory biomarkers will be necessary to determine whether CBC-derived inflammatory variability has clinically meaningful relevance in schizophrenia research.

## 5. Conclusions

The present study provides an exploratory panel-based characterization of CBC-derived inflammatory index distributions in hospitalized patients with schizophrenia using routinely available hematological parameters. Despite the absence of patient-level identifiers and longitudinal linkage, the findings demonstrated substantial variability in inflammatory marker distributions across the analyzed panel-level dataset, particularly for NLR, PLR, and SII.

Exploratory clustering analyses additionally suggested that a subset of panels exhibited relatively elevated inflammatory marker distributions compared with the broader dataset. However, because clustering procedures were performed within an exploratory unsupervised analytical framework and major clinical confounding variables were unavailable, these observations should not be interpreted as evidence of clinically validated inflammatory phenotypes or biologically discrete schizophrenia subtypes.

The present findings are generally consistent with the broader literature suggesting that inflammatory variability may contribute to biological heterogeneity within schizophrenia-related populations [21,26,27,31,32]. Nevertheless, the current analyses remain descriptive and hypothesis-generating in nature because the dataset lacked healthy control comparisons, longitudinal clinical outcomes, symptom severity measures, treatment response variables, and integrated molecular inflammatory biomarkers.

Within these limitations, routinely available CBC-derived inflammatory indices may still provide a practical and scalable framework for exploratory investigation of inflammatory variability patterns in real-world psychiatric datasets. Future studies integrating longitudinal clinical follow-up, cytokine profiling, neuroimaging measures, cognitive assessments, and multimodal biological datasets will be necessary to determine whether hematological inflammatory variability has clinically meaningful relevance in schizophrenia research and whether such patterns correspond to reproducible and reproducible patterns of inflammatory variability within schizophrenia-related populations.

## Figures and Tables

**Table 1:** Descriptive distributional characteristics of hematological variables and CBC-derived inflammatory indices within the analyzed panel-level schizophrenia dataset. Data are presented as median (IQR) or *n* (%). Abbreviations: WBC, white blood cell count; NEUT#, absolute neutrophil count; LYM#, absolute lymphocyte count; MONO#, absolute monocyte count; PLT, platelet count; RDW-CV, red cell distribution width coefficient of variation; MPV, mean platelet volume; NLR, neutrophil-to-lymphocyte ratio; MLR, monocyte-to-lymphocyte ratio; PLR, platelet-to-lymphocyte ratio; SII, systemic immune–inflammation index. Data are presented at the panel level rather than the patient level. Repeated observations originating from the same individual could not be excluded because patient-level identifiers were unavailable.

Variable	Median (IQR) or n (%)
Sex distribution (panel-level observations)	Male: 525 (61.2%); Female: 333 (38.8%)
Panel-level age (years)	32 (25–41)
WBC	8.73 (7.22–10.58)
NEUT#	5.55 (4.41–7.28)
LYM#	2.18 (1.70–2.66)
MONO#	0.54 (0.42–0.66)
PLT	270.00 (226.25–326.00)
RDW-CV	13.40 (12.80–14.40)
MPV	10.10 (9.40–11.00)
NLR	2.51 (1.95–3.55)
MLR	0.25 (0.19–0.31)
PLR	124.10 (100.40–163.94)
SII	686.96 (484.81–1045.85)

**Table 2:** Distribution of CBC-derived inflammatory indices across predefined exploratory threshold categories within the analyzed panel-level schizophrenia dataset. Data are presented as n (%). Threshold categories were used for descriptive exploratory characterization only and should not be interpreted as clinically validated schizophrenia-specific inflammatory cut-off values. Data are presented at the panel level rather than the patient level. Repeated observations originating from the same individual could not be excluded because patient-level identifiers were unavailable.

Index/Parameter	Exploratory Threshold Category	n (%)
NLR	>3	308 (35.9%)
NLR	>5	99 (11.5%)
PLR	>150	274 (31.9%)
PLR	>300	21 (2.5%)
SII	>500	624 (72.7%)
SII	>1000	232 (27.0%)
RDW-CV	>14.5	188 (21.9%)
MPV	>11	196 (22.8%)

**Table 3:** Exploratory clustering characteristics of CBC-derived inflammatory indices within the analyzed panel-level schizophrenia dataset. Cluster labels were assigned descriptively according to relative inflammatory marker distributions and should not be interpreted as clinically validated or biologically definitive inflammatory phenotypes. Data are presented at the panel level rather than the patient level. Repeated observations originating from the same individual could not be excluded because patient-level identifiers were unavailable. Abbreviations: NLR, neutrophil-to-lymphocyte ratio; MLR, monocyte-to-lymphocyte ratio; PLR, platelet-to-lymphocyte ratio; SII, systemic immune–inflammation index; WBC, white blood cell count; PLT, platelet count.

Cluster	Descriptive Cluster Label	n (%)	NLR (Median)	MLR (Median)	PLR (Median)	SII (Median)	WBC (Median)	PLT (Median)
0	Lower inflammatory marker distribution	715 (83.3%)	2.30	0.23	116.95	626.21	8.42	266
1	Relatively elevated inflammatory marker distribution	143 (16.7%)	5.62	0.40	222.39	1746.71	10.66	310

## Data Availability

The datasets generated and/or analyzed during the current study are available from the corresponding author on reasonable request.

## Abbreviations

AUC	Area Under the Curve
BASO#	Absolute Basophil Count
BASO%	Basophil Percentage
BMI	Body Mass Index
CBC	Complete Blood Count
CRP	C-Reactive Protein
EOS#	Absolute Eosinophil Count
EOS%	Eosinophil Percentage
FDR	False Discovery Rate
Hb	Hemoglobin
Hct	Hematocrit
IQR	Interquartile Range
LYM#	Absolute Lymphocyte Count
LYM%	Lymphocyte Percentage
MCV	Mean Corpuscular Volume
MLR	Monocyte-to-Lymphocyte Ratio
MONO#	Absolute Monocyte Count
MONO%	Monocyte Percentage
MPV	Mean Platelet Volume
NEUT#	Absolute Neutrophil Count
NEUT%	Neutrophil Percentage
NLR	Neutrophil-to-Lymphocyte Ratio
PCT	Plateletcrit
PDW	Platelet Distribution Width
PLR	Platelet-to-Lymphocyte Ratio
PLT	Platelet Count
P-LCR	Platelet Large Cell Ratio
RBC	Red Blood Cell Count
RDW-CV	Red Cell Distribution Width Coefficient of Variation
RDW-SD	Red Cell Distribution Width Standard Deviation
ROC	Receiver Operating Characteristic
SII	Systemic Immune–Inflammation Index
WBC	White Blood Cell Count
